# Antisecretory factor is safe to use as add-on treatment in newly diagnosed glioblastoma

**DOI:** 10.1186/s12883-023-03119-4

**Published:** 2023-02-18

**Authors:** Erik Ehinger, Jan Kopecky, Anna Darabi, Edward Visse, Charlotte Edvardsson, Gregor Tomasevic, David Cederberg, Mattias Belting, Johan Bengzon, Peter Siesjö

**Affiliations:** 1 Neurosurgery, Department of Clinical Sciences, Lund University, Skåne University Hospital, Lund, Sweden; 2 Glioma Immunotherapy Group, Neurosurgery, Department of Clinical Sciences, Lund University, Lund, Sweden; 3 Oncology, Department of Clinical Sciences, Lund University, Skåne University Hospital, Lund, Sweden; 4 Department of Immunology, Genetics and Pathology, Science for Life Laboratory, Uppsala University, Uppsala, Sweden; 5 Department of Hematology, Oncology and Radiophysics, Skåne University Hospital, Lund, Sweden; 6 Lund Stem Cell Center, Department of Clinical Sciences, Lund University, Lund, Sweden

**Keywords:** Glioblastoma, Antisecretory factor, Novel treatments against glioblastoma, Salovum

## Abstract

**Purpose:**

Glioblastoma (GBM) is the most common primary malignant brain tumor in adults. Despite the best available treatment, prognosis remains poor. Current standard therapy consists of surgical removal of the tumor followed by radiotherapy and chemotherapy with the alkylating agent temozolomide (TMZ). Experimental studies suggest that antisecretory factor (AF), an endogenous protein with proposed antisecretory and anti-inflammatory properties, may potentiate the effect of TMZ and alleviate cerebral edema. Salovum is an egg yolk powder enriched for AF and is classified as a medical food in the European Union. In this pilot study, we evaluate the safety and feasibility of add-on Salovum in GBM patients.

**Methods:**

Eight patients with newly diagnosed, histologically confirmed GBM were prescribed Salovum during concomitant radiochemotherapy. Safety was determined by the number of treatment-related adverse events. Feasibility was determined by the number of patients who completed the full prescribed Salovum treatment.

**Results:**

No serious treatment-related adverse events were observed. Out of 8 included patients, 2 did not complete the full treatment. Only one of the dropouts was due to issues directly related to Salovum, which were nausea and loss of appetite. Median survival was 23 months.

**Conclusions:**

We conclude that Salovum is safe to use as an add-on treatment for GBM. In terms of feasibility, adherence to the treatment regimen requires a determined and independent patient as the large doses prescribed may cause nausea and loss of appetite.

**Trial registration:**

ClinicalTrials.gov NCT04116138. Registered on 04/10/2019.

**Supplementary Information:**

The online version contains supplementary material available at 10.1186/s12883-023-03119-4.

## Introduction

Glioblastoma (GBM) is the most common malignant primary tumor of the brain. Despite our best efforts, prognosis remains dismal with a median survival of 7–18 months [1]. Current standard therapy consists of surgical resection followed by radiotherapy and chemotherapy with the alkylating agent temozolomide (TMZ) [2, 3].

Several different mechanisms are thought to contribute to the therapy resistance of GBM. The infiltrative growth pattern of GBM makes surgical removal of all tumor cells impossible. Although surgical methods have been refined over the years, the challenge ahead lies not in surgical advancements, but in developing novel oncological or multidisciplinary therapies. An issue when exploring new ways to treat GBM is the heterogeneity of the disease, not only between different individuals, but also between regions within the tumor itself [4]. This intratumoral heterogeneity, which has been attributed to both the presence of cancer stem cells and dynamic epigenetic mechanisms, might explain the poor prognosis and imminent recurrence of GBM; the tumor consists of different cellular populations which each might respond differently to therapy. Thus, there is no single druggable target, and the multitude of oncogenic pathways contribute to treatment resistance. The GBM tumor microenvironment (TME) constitutes a complicated array of cellular populations and tumor-supporting mechanisms. Glioblastoma cells manipulate and recruit non-transformed cells to produce an advantageous TME for tumor growth. Innate and adaptive immune cells are modified to support tumor growth and to suppress an antitumor immune response. Crosstalk between these cell populations through complex signaling pathways contribute to tumor growth, angiogenesis, invasion, immunosurveillance escape and therapy resistance [5].

In addition to multidrug resistance, GBM, as most solid tumors, displays an elevated intratumoral interstitial fluid pressure (IFP) [6, 7]. The increased IFP disturbs blood flow within the TME, thereby inducing hypoxia as a driving force of angiogenesis and cancer progression. Elevated IFP may also provide a barrier for drug uptake to the tumor and by this mechanism further contribute to therapy resistance [7].

Antisecretory factor (AF) is an endogenous and essential protein with proposed antisecretory and anti-inflammatory properties [8]. There are currently two approved methods of increasing antisecretory factor levels in humans and animals: SPC-flakes (specially processed cereals) and Salovum. SPC-flakes consist of oats subjected to malting, a hydrothermal treatment process. Consuming SPC-flakes stimulates endogenous production of AF [9]. Salovum is a freeze-dried egg yolk powder based on eggs from hens fed with SPC-flakes and contains large amounts of AF [10]. Salovum and SPC-flakes are classified as medical food by the European Union and can be bought without a prescription in pharmacies in Sweden. Salovum has been used in clinical trials for a variety of conditions, including diarrheal diseases [11–13], inflammatory bowel disease [9, 14, 15], Ménière’s disease [16–20] and traumatic brain injury [21, 22]. No adverse effects have been reported, even in high doses [13, 15, 23].

AF has been shown to reduce the elevated IFP in experimental models of solid tumors [24]. Ilkhanizadeh et al. demonstrated that exogenous administration of AF reduced IFP-levels, increased drug uptake to the tumor and inhibited tumor growth in GBM-xenografted mice [25]. They also showed that SPC-flakes combined with TMZ successfully prevented tumor growth and increased overall survival at 120 days from 30 to 100% compared to TMZ monotherapy [25]. Our own results show that intratumoral delivery of an active AF peptide, AF-16, boosts the effect of intratumoral chemotherapy and immune reactivity in an experimental glioma model [26].

Glucocorticoids (GCs) play a major role in the clinical management of glioblastoma. In clinical practice, GCs are prescribed liberally to relieve neurological symptoms and brain edema produced by the tumor. There is, however, increasing evidence that GCs may have a negative impact on survival in GBM patients [27]. Mechanistically, it has been proposed that GCs induce a gene signature in the tumor that contribute to a worse prognosis [28]. Long-term therapy with GCs is also associated with many unwanted side effects such as diabetes, myopathy, insomnia, anxiety, and infections. Salovum has recently been shown to reduce increased intracranial pressure in two pilot studies on patients with traumatic brain injury [21, 22]. We hypothesize that Salovum, by reducing tumoral IFP, might both alleviate the peritumoral edema in GBM and facilitate temozolomide delivery to the tumor.

Here, we investigate the safety, feasibility and survival of add-on Salovum in patients with newly diagnosed GBM undergoing concomitant radiochemotherapy. We also assess if high doses of Salovum can contribute to reduced corticosteroid dependency. As Salovum consists of dried egg yolk, blood levels of lipids were monitored during treatment.

## Methods

### Primary aim

The primary aim was to assess the safety and feasibility of add-on Salovum during concomitant radiochemotherapy in patients with newly diagnosed GBM. Number of adverse events (CTCAE version 5) and cholesterol levels were used as safety parameters. The ratio of patients completing the full trial was used to assess feasibility.

### Secondary aim

Secondary aims were overall survival (OS), progression-free survival (PFS) and to assess if Salovum can contribute to a reduced corticosteroid dependency in GBM patients.

### Ethical permit

This study was reviewed and approved by the Swedish Ethical Review Authority, no. 2019–03781, in accordance with the ethical standards of the Declaration of Helsinki. Written informed consent to participate in this study was provided by all patients.

### Study design and patients

This was a prospective, open-label pilot study. Eight patients aged 51–62 with newly diagnosed GBM were recruited at the neurosurgical department at Skåne University Hospital between September 2019 and February 2020. Included patients underwent surgical resection of the tumor followed by concomitant radiochemotherapy and adjuvant chemotherapy. Salovum was administered during concomitant treatment. Written consent was obtained. Egg allergy was an exclusion criterion, however no patients declared to have this.

### Controls

To compare survival, a retrospective cohort was collected consisting of all glioblastoma patients treated at our center in the years 2016–2017. To minimize confounding, we matched controls for age and included only patients who had undergone surgical resection followed by concomitant and adjuvant therapy.

### Treatment

#### Standard treatment (Stupp regimen)

External fractionated RT was delivered in 30 fractions at 2 Gy per fraction, 5 days a week for 6 weeks. Concomitant chemotherapy consisted of TMZ at a dose of 75 mg/m^2^, administered 7 days per week from the first day of RT until the last, for a total of 40 days. After a four-week break, patients received adjuvant TMZ at a daily dose of 150–200 mg/m^2^ for 5 days in six cycles of 28 days.

#### Trial treatment

**Fig. 1:**
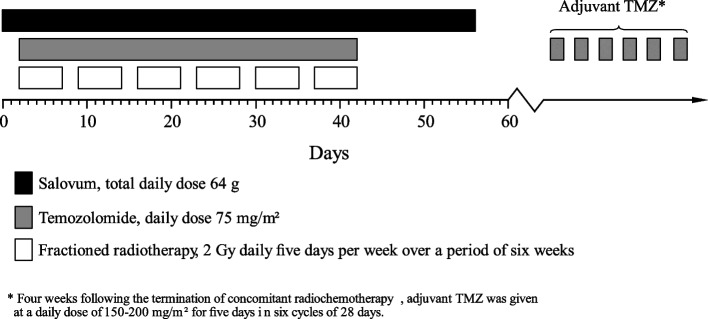
Schematic overview of our treatment regimen. Day 0 is defined as start of Salovum therapy, two days before radiation start

The egg yolk powder, Salovum, was dissolved in an unheated liquid of the patients’ choice and was administered orally four times daily at a total daily dose of 64 g. Salovum therapy was initiated 2 days prior to the start of RT and administered daily until 14 days after termination of the concomitant radiochemotherapy, for a total of 56 days. Figure 1 shows a schematic overview of the regimen.

### Patient evaluation and follow-up

Patients underwent clinical evaluations before, during and after Salovum therapy. Cognitive function was assessed with the Mini Mental State Examination (MMSE) [29] to determine preoperative status. The Eastern Cooperative Oncology Group (ECOG) performance status was noted [30]. MRI was performed before and after Salovum therapy, before cycles 3 and 6 of adjuvant temozolomide and at 12 months. After the first year, MRI was performed every 3 months until 24-month follow-up. Overall survival was defined as time from first surgery to death. Progression-free survival was defined as time from first surgery to progression confirmed by consensus at a multidisciplinary conference.

### Blood analysis and molecular diagnostics

Due to the high intake of egg yolk during the trial hypercholesterolemia might be induced and cholesterol was analyzed according to local laboratory standards. IDH analysis was conducted by multiplex ligation probe amplification or qPCR, dependent on the clinical scenario and if co-analysis of 1p19q loss of heterozygosity was performed.

### Corticosteroid dependency

Postoperatively, an attempt was made to discontinue corticosteroids according to a 10-day tapering schedule. Successful cessation was noted in the baseline assessment. The current daily corticosteroid dose was registered every week of Salovum treatment. Daily betamethasone doses of less than 2 mg were defined as no steroids (physiological dose). Treating physicians were informed that discontinuation or dose-reduction of corticosteroids was to be attempted throughout the course of the trial.

### Statistical analyses

Survival analysis from operation date to death was plotted according to the Kaplan-Meier method. Patients and controls were censored at latest follow-up, February 2022. A log-rank test was used to compare survival between study patients and controls. Blood lipids were compared using a paired Wilcoxon Signed-Ranks Test. All statistical analyses were performed using free statistical software, R version 4 (https://www.R-project.org/).

## Results

### Patients

Eight out of ten eligible patients were included while 2/10 were excluded due to withdrawal of consent and death before baseline. Supplementary fig. S1 shows an inclusion flowchart. Baseline patient characteristics, cumulative betamethasone doses and tumor status at last follow-up are presented in Table 1. Median age was 57.5 years (range 51–62). All patients had an ECOG performance status ≤1. After surgery, 3 patients were considered to have undergone gross total resection and 5 had residual tumor (mean residual tumor volume 4 ml, range 0,1–11,5 ml). All tumors were IDH wildtype, and two patients had tumors with methylated O^6^-methylguanine DNA methyltransferase (MGMT) promoter regions.

**Table 1:** Baseline patient characteristics, cumulative steroid doses and time from first operation to recurrence or death (or time to last follow-up if still alive or recurrence-free)

Pat	Degree of completion (reason for withdrawal)	Age	Sex	MGMT status	WHO/ECOG performance status	MMSE score	Preoperative tumor volume (ml)
#1	Full	57	M	Unmethylated	1	29	9.2
#2	21/56 days (pulmonary embolism)	55	M	Unmethylated	1	28	45.7
#3	22/56 days (nausea)	62	M	Unmethylated	1	28	30.6
#4	Full	59	M	Unmethylated	1	28	40.5
#5	Full	51	M	Methylated	1	27	48.4
#6	Full	58	M	Unmethylated	1	28	30.2
#7	Full	54	M	Methylated	1	29	2.7
#8	Full	59	M	Unmethylated	0	28	6.7
Pat	Gross total resection on postoperative MRI	Residual tumor on postoperative MRI (ml)	Steroids at baseline? (dose, mg)	Cumulative betamethasone during trial (mg)	Repeat surgery? (months from primary op)	Cycles of adjuvant TMZ	
#1	N	0.55	N (0)	5	N	0	
#2	Y	0	Y (2)	23	Y (13.1)	6	
#3	Y	0	N (1.5)	14	Y (8)	5	
#4	N	7.3	Y (2)	143	N	5	
#5	N	0.1	Y (2)	117	N	6	
#6	N	11.5	N (1)	135	Y (8.7)	5	
#7	Y	0	N (0)	96	Y (21.2)	6	
#8	N	0.6	N (0)	77	Y (20.7)	6	
Pat	Tumor treating fields?	Overall survival (months)	Progression free survival (months)				
#1	N	12.4	4.3				
#2	Y	22.3	12.6				
#3	N	24.9	7.6				
#4	N	11.3	6.5				
#5	N	26^a^	26^a^				
#6	Y	13.8	7.8				
#7	Y	24^a^	20.3				
#8	Y	23.6	18.6				

^a^Alive or recurrence-free at last follow-up

### Safety and feasibility

Of the 8 included patients, 6 completed full trial treatment. One patient had a pulmonary embolism after half of the concomitant treatment and had to discontinue TMZ/Salovum. One patient withdrew after half of the trial because of nausea and loss of appetite (grade 1 according to CTCAE) following Salovum intake. No other treatment-related adverse events were observed during the Salovum treatment. No hematologic toxicity was observed during Salovum therapy. At the end of the concomitant treatment, ECOG performance score remained unchanged for all patients but one who declined due to early progression. In summary 6/8 patients could complete the prescribed dose, and no safety issues were identified.

### Lipids

Cholesterol levels in blood were compared before, during and after Salovum therapy (median 6.3, 6.2 and 5.8, respectively). A paired Wilcoxon signed-rank test indicated that this difference was not statistically significant (*p* = 0.4, before vs. during) (*p* = 0.96, before vs. after).

### Survival

#### Study patients

**Fig. 2:**
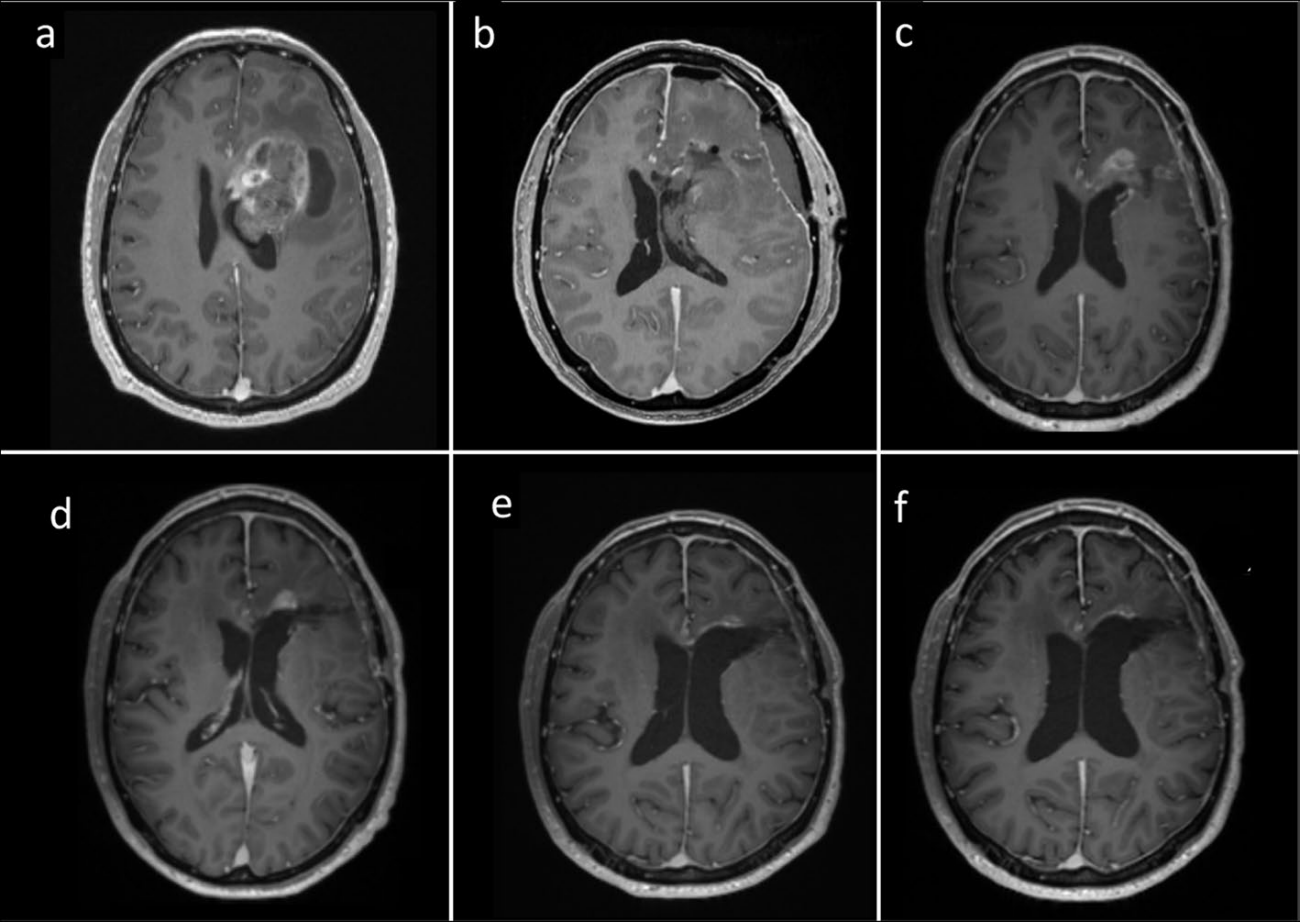
Contrast enhanced MRI studies of one patient (#5) **a** before surgery, **b** postoperatively, **c** after completed Salovum therapy, **d** at 12-month follow-up, **e** at 18-month follow-up and **f** at 24-month follow-up. This patient displayed increased contrast enhancement after the concomitant therapy followed by diminishing enhancement and durable tumor control, possibly due to initial radionecrosis followed by delayed immunologic response to therapy

At the latest follow-up, three (38%) patients remained alive, one of whom was free from recurrence. Figure 2 illustrates this latter patient. Survival data are reported in Table 1. Median OS was 23.0 months. Median PFS was 10.2 months.

#### Retrospective controls

For retrospective controls, 28 age-matched and treatment-matched GBM-patients were identified. All controls had gone through surgical resection followed by concomitant and adjuvant treatment. Median age at diagnosis was 55 years (range 50–62). Sixty-four percent were male. Median OS was 14.8 months. Median PFS was 9.4 months. Characteristics and data of controls are reported in Table 2. Molecular and genetic diagnostics of many tumors were missing because these analyses were not performed routinely at the time.

**Table 2:** Characteristics of retrospective controls and treated patients

Characteristic	Retrospective control group (*N* = 28)	Salovum group (*N* = 8)
Age – yr (range)		
Median	55.5 (50–62)	57.5 (51–62)
Gender – no. (%)		
Male	18 (64)	8 (100)
Female	10 (36)	0
WHO/ECOG performance status – no. (%)		
0	18 (64)	7 (88)
1	6 (21)	1 (13)
2	3 (11)	0
3	1 (4)	0
Surgical resection – no. (%)	28 (100)	8 (100)
GTR	13 (46)	3 (38)
MGMT status – no. (%)		
Methylated	4 (14)	2 (25)
Unmethylated	6 (21)	6 (75)
Unknown	18 (64)	0
IDH status – no. (%)		
Wildtype	19 (68)	8 (100)
Mutated	0	0
Unknown	9 (32)	0
Survival time – no. (%)		
< 12 mo	7 (25)	1 (13)
12–18 mo	11 (39)	2 (25)
18–24 mo	4 (14)	1 (13)
> 24 mo	6 (21)	4 (50)
Median – mo (range)	14.8 (3.4–60.6)	23.0 (11.3–26.0)
Time from diagnosis to recurrence – no. (%)		
< 6 mo	8 (29)	1 (13)
6–12 mo	13 (46)	3 (38)
≥ 12 mo	6 (21)	4 (50)
Median – mo (range)	9.4 (3.4–29.1)	10.2 (4.3–26.0)
Full radiotherapy completed – no. (%)	27 (96)	8 (100)
Adjuvant TMZ cycles completed – no. (%)		
6	16 (57)	4 (50)
3–5	8 (29)	3 (38)
< 3	4 (14)	1 (13)
Second line treatment – no. (%)		
Lomustine	12 (43)	4 (50)
TMZ	4 (14)	1 (13)
None	12 (43)	3 (38)

**Fig. 3:**
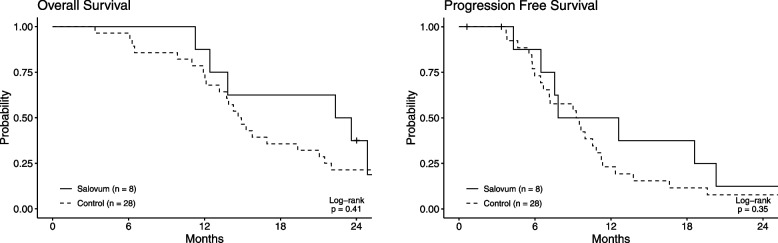
Overall survival as illustrated by Kaplan-Meier graphs

Kaplan-Meier curves of the first 24 postoperative months are shown in Fig. 3. Patients still alive or free from recurrence were censored at latest follow-up. No statistically significant difference was found between trial patients and controls regarding OS (*p* = 0.43) or PFS (*p* = 0.35) using a log-rank test.

### Corticosteroids

**Fig. 4:**
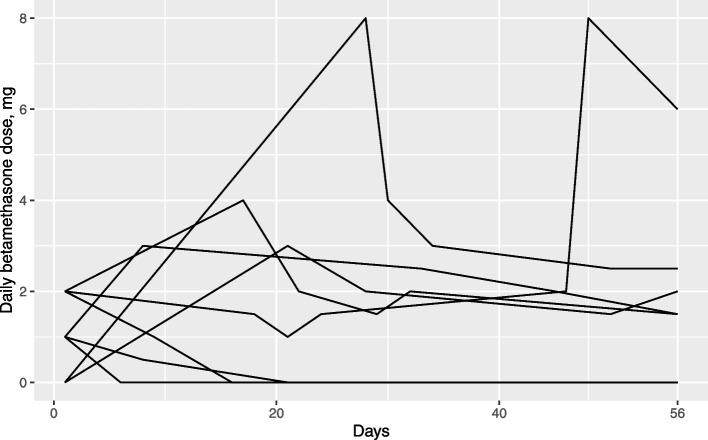
Daily betamethasone doses during the concomitant radiochemotherapy and Salovum treatment. Each line represents one patient

Individual betamethasone doses during the trial are presented in Fig. 4. Three (3/8) patients had a complete reduction of steroid dose. Four (4/8) patients had an initial increase in dose followed by a sustained dose reduction. One (1/8) patient had to increase dose during treatment and could not taper. Only one patient had a daily betamethasone dose above 2,5 mg at the end of the trial.

## Discussion

Glioblastoma is an incurable disease despite major research efforts in the last decades. Here we show that add-on treatment with the AF-enriched dietary supplement Salovum is safe and feasible for patients with GBM undergoing concomitant radiochemotherapy.

### Safety

Based on previous use in and outside clinical trials for various diagnoses, Salovum can be considered as safe. However, this is the first trial in which Salovum is given to humans in combination with TMZ and RT as treatment for GBM. As experimental data indicate that AF-16, SPC and Salovum can potentiate chemotherapy with temozolomide, it cannot be excluded that the combination of AF and TMZ could also aggravate side effects of temozolomide, with a potential risk of toxicity due to extensive cell death. However, during the concomitant phase of treatment, we observed no such events.

### Feasibility

Only one patient had to discontinue therapy because of an issue directly related to Salovum. This patient reported nausea and loss of appetite following intake, attributed to the bad taste and unfavorable consistency of the Salovum drink. Due to issues with bad taste and loss of appetite patients need a well-preserved cognitive function to be compliant with the regimen. This may provide a self-selection bias when evaluating survival in the upcoming randomized clinical trial, because patients with a well-preserved cognitive function may have a better prognosis from the outset [31].

The choice of dosage of Salovum in this trial was in part arbitrary and based on former studies with a documented treatment effect of Salovum in childhood diarrhea [11, 13]. In these studies, patients were given Salovum either in a single dose of 8-16 g, or 8 g every 5 h until recovery. Here, we administer 16 g Salovum four times daily for several weeks, which amounts to very large doses. The loss of appetite and nausea experienced by patients may be attributed to the caloric load of Salovum, but also to bad taste from ingesting cold egg yolk powder. To improve feasibility in future studies, we will consider lowering dosage. We will also consider adding Salovum to the adjuvant temozolomide cycles, where patients would ingest Salovum during treatment, i.e. 6–8 days a month.

### Survival

It is a well-known issue that GBM-patients included in clinical trials constitute a highly selected group with better prognosis than the general GBM population [32]. This is presumably because more than half of newly diagnosed GBM-patients do not fulfil the inclusion criteria of most clinical trials, including our study. To make a meaningful comparison of survival, we selected an age and treatment matched retrospective cohort. Compared to this cohort, the treated patients displayed higher median OS (23.0 months versus 14.8 months) and PFS (10.2 months versus 9.4 months) compared to controls, but this was not statistically significant.

Efficacy of TMZ therapy depends on the reparative ability of the tumor cells, which is in part controlled by the DNA repair protein MGMT [33]. MGMT promoter methylation status is one of the few clinically available prognostic factors for GBM. As the effect of TMZ on survival is predominantly associated with tumors with methylated MGMT, we hypothesize that the effect of Salovum would be most pronounced against MGMT-methylated tumors. Figure 2 illustrates one of the study patients with methylated MGMT, who displayed excellent response to therapy and remains free from recurrence 26 months after diagnosis.

### Steroids

Management of corticosteroid dosage to GBM patients is conducted according to the individual treating physician’s judgement. Although the EANO guidelines advise tapering after surgery, there is no direct guidance regarding the timing of how to conduct the tapering [34]. There is also increasing evidence that corticosteroid use in GBM is associated with a worse prognosis [35], and corticosteroid use during radiochemotherapy is an independent predictor of poor outcome in GBM, even when accounting for the confounding factor that higher corticosteroid usage is often coupled with larger tumors and less extent of tumor resection [27]. There is consequently an impetus to investigate alternatives for edema reduction in GBM lacking the detrimental effects of high dose steroid medication. In the present study, corticosteroids could not be discontinued in all patients during or after administration of Salovum but only 1/8 patients had a dose higher than 2.5 mg betamethasone at the end of concomitant treatment. Most patients were also able to reduce their doses over time during the trial. Interestingly, one of our patients experienced a worsening of neurological symptoms just days after ceasing with Salovum and had to start steroid treatment. In conclusion, administration of Salovum might reduce the use of steroids but this must be proven in a randomized trial.

### Possible mechanisms of AF

The cellular and molecular mechanisms by which AF operate in the brain are poorly understood. Antisecretory factor regulates the transport of water and ions across cellular membranes, and was initially studied for its antisecretory properties [36]. Later, AF was also demonstrated to have immunoregulatory properties [37]. Antisecretory factor is expressed by many cells, including macrophages and cells in the brain perivascular space [37]. Davidson et al. demonstrated that targeting AF with antibodies enhanced the T-cell response in vitro, suggesting that AF plays an immunoregulatory role. Also, it has been proposed that AF may be involved in the complement cascade of the innate immune system [38]. Most recently, a study of AF16, an active peptide from the AF protein, showed that AF16 increased the secretion of typically proinflammatory cytokines from human and mouse M0 macrophages, and that intratumoral co-administration of TMZ and AF16 resulted in synergistically increased survival in glioma-bearing mice [26]. Taken together, the antisecretory and immunomodulatory mechanisms exerted by AF may contribute to suppressing tumor progression by reducing tumor-promoting inflammation and lowering tumoral IFP, as well as giving symptomatic relief by reducing brain edema. This is however still speculative and further research is needed to confirm these hypotheses.

### Limitations

This was an open label phase 1 trial conducted in a small number of patients. The study was designed to evaluate safety and feasibility and as such presents with limitations when assessing secondary outcomes. Although a trend towards better survival might be discerned here, the trial was not designed to specifically evaluate treatment effects. Efficacy will be further evaluated in an upcoming randomized clinical trial. The data is too limited to draw any conclusions about changes in cholesterol levels from Salovum usage but there was no obvious increase during treatment. Although our patients had generally low corticosteroid doses, it is from our limited data difficult to conclude whether Salovum can contribute to reduced corticosteroid dependency.

## Conclusions

Salovum is safe to use as an add-on treatment for GBM. In terms of feasibility, compliance with the treatment regimen requires a determined and independent patient because the large doses prescribed may cause nausea and loss of appetite. To address this issue, we propose a dose reduction of Salovum in future clinical trials. Whether Salovum affects cholesterol levels, survival or can contribute to a reduced corticosteroid dependency remain uncertain but will be further investigated in an upcoming randomized clinical trial.

## Supplementary Information

**Additional file 1: Supplementary figure S1.** Flowchart of inclusion and protocol compliance.

## Data Availability

The datasets used and analyzed in the current study are available from the corresponding author on reasonable request.
