# Supplementary material for: Antisecretory factor is safe to use as add-on treatment in newly diagnosed glioblastoma
Source: BMC Neurol. 2023 Feb 18;23:76. doi: 10.1186/s12883-023-03119-4 (PMC9938624; doi:10.1186/s12883-023-03119-4)
Supplement: Supplementary file 1 — Additional file 1: Supplementary figure S1. Flowchart of inclusion and protocol compliance. [file 12883_2023_3119_MOESM1_ESM.docx]

Supplementary figure S1. Flowchart of inclusion and protocol compliance.

Eligible patients

(n = 10)

Excluded

Withdrawal of consent (n = 1)

Deceased prior to baseline (n = 1)

Included patients

(n = 8)

Completed full treatment of 56 days

(n = 6)

Partial treatment

Completed 21/56 days (n = 1)

Completed 22/56 days (n = 1)
